# TLR4 Participates in the Inflammatory Response Induced by the AAF/II Fimbriae From Enteroaggregative *Escherichia coli* on Intestinal Epithelial Cells

**DOI:** 10.3389/fcimb.2019.00143

**Published:** 2019-05-03

**Authors:** Alejandra Alvestegui, Mauricio Olivares-Morales, Ernesto Muñoz, Rachel Smith, James P. Nataro, Fernando Ruiz-Perez, Mauricio J. Farfan

**Affiliations:** ^1^Departamento de Pediatría, Facultad de Medicina, Centro de Estudios Moleculares, Hospital Dr. Luis Calvo Mackenna, Universidad de Chile, Santiago, Chile; ^2^Department of Pediatrics, University of Virginia School of Medicine, Charlottesville, VA, United States

**Keywords:** enteroaggregative *E. coli*, Aggregative adherence fimbriae, inflammation, toll-like receptors, interleukin-8

## Abstract

Enteroaggregative *Escherichia coli* (EAEC) infections are one of the most frequent causes of persistent diarrhea in children, immunocompromised patients and travelers worldwide. The most prominent colonization factors of EAEC are aggregative adherence fimbriae (AAF). EAEC prototypical strain 042 harbors the AAF/II fimbriae variant, which mediates adhesion to intestinal epithelial cells and participates in the induction of an inflammatory response against this pathogen. However, the mechanism and the cell receptors implicated in eliciting this response have not been fully characterized. Since previous reports have shown that TLR4 recognize fimbriae from different pathogens, we evaluated the role of this receptor in the response elicited against EAEC by intestinal cells. Using a mutual antagonist against TLR2 and TLR4 (OxPAPC), we observed that blocking of these receptors significantly reduces the secretion of the inflammatory marker IL-8 in response to EAEC and AAF/II fimbrial extract in HT-29 cells. Using a TLR4-specific antagonist (TAK-242), we observed that the secretion of this cytokine was significantly reduced in HT-29 cells infected with EAEC or incubated with AAF/II fimbrial extract. We evaluated the participation of AAF/II fimbriae in the TLR4-mediated secretion of 38 cytokines, chemokines, and growth factors involved in inflammation. A reduction in the secretion of IL-8, GRO, and IL-4 was observed. Our results suggest that TLR4 participates in the secretion of several inflammation biomarkers in response to AAF/II fimbriae.

## Introduction

Enteroaggregative *Escherichia coli* (EAEC) strains form a heterogeneous group of enteric pathogens that has been associated with both acute and persistent diarrhea in children, immunocompromised patients, and travelers worldwide (Huang et al., 2006; Hebbelstrup et al., 2014).

The infective cycle of EAEC begins with bacterial adhesion to the intestinal epithelium, followed by enterotoxin and cytotoxin secretion and the induction of an inflammatory response (Estrada-Garcia and Navarro-Garcia, 2012). EAEC strains can be divided into typical and atypical strains depending on the presence or absence of the plasmid-encoded virulence regulator AggR. This master regulator controls the expression of several chromosome- and plasmid-encoded genes, including aggregative adherence fimbriae (AAFs), a key colonization factor in EAEC pathogenesis (Harrington et al., 2006; Morin et al., 2013). To date, five variants of AAF have been described: AAF/I, AAF/II, AAF/III, AAF/IV, and AAF/V (Nataro et al., 1992; Czeczulin et al., 1997; Bernier et al., 2002; Boisen et al., 2008).

Prototype EAEC strain 042 harbors the AAF/II variant, which has been shown to mediate adhesion to cytokeratin-8 and several extracellular matrix proteins such as collagen, laminin and fibronectin (Farfan et al., 2008; Izquierdo et al., 2014a,b). Additionally, AAF/II fimbriae have been shown to contribute to the inflammatory response elicited by EAEC in colonic epithelial cells (Harrington et al., 2005; Boll et al., 2012) and the migration of neutrophils across the epithelium mediated by MUC1 mucin (Boll et al., 2017). However, the molecular mechanism involved in the inflammatory response to these fimbriae and the receptors associated with this process are unknown.

Toll-like receptors (TLRs) are a family of transmembrane immune receptors that trigger an inflammatory activation in response to pathogen-associated molecular patterns (PAMPs) (Lee et al., 2004; Takeda and Akira, 2004). Currently, ten TLRs have been described, of which agonists for nine of them have been determined. Fimbriae recognition by TLRs has been described previously. TLR2 is involved in the recognition of fimbriae from *Porphyromonas gingivalis* and the curli from *Salmonella enterica*; TLR4 recognizes types I and P fimbriae from uropathogenic *E. coli* (Asai et al., 2001; Tukel et al., 2005; Sirard et al., 2006; Davey et al., 2008; Mossman et al., 2008).

Given the above, we aimed to evaluate the participation of TLR4 in the inflammatory response induced by AAF/II of EAEC strain 042. Our results implicate TLR4 in the inflammatory response induced by AAF/II fimbriae on intestinal epithelial cells.

## Materials and Methods

### Bacterial Strains

Prototype EAEC strain 042 (O44:H18) was originally isolated from a child with diarrhea in Lima, Peru. Strain 042Δ*fliC* is an aflagellar mutant that harbors the suicide plasmid pJP5603 inserted into the *fliC* gene (Steiner et al., 2000). Bacteria were grown overnight under static conditions in Luria-Bertani (LB) broth and then subcultured in DMEM-HG (Gibco, Life Technologies) for expression of the virulent phenotype.

### Cell Lines

T84 cells (ATCC CCL-248) were routinely maintained in DMEM F-12 supplemented with 10% fetal bovine serum (FBS), streptomycin (10 μg/mL) and penicillin (10 U/mL) at 37°C under 5% CO_2_. HT-29 cells (ATCC HTB-38) were maintained in McCoy's 5A medium with 10% FBS, streptomycin (10 μg/mL) and penicillin (10 U/mL) at 37°C under 5% CO_2_.

### Fimbriae Extraction

AAF/II fimbriae from EAEC strains were purified as previously described (Izquierdo et al., 2014a). Briefly, EAEC strain 042Δ*fliC* was grown in 1 L DMEM/HG medium (Gibco, Life Technologies) at 37°C with shaking until an OD_600_ of 1.0. Bacteria were collected by centrifugation at 4,000 × *g* for 25 min and resuspended in 10 mL of 0.5 mM Tris 75 mM NaCl solution. The bacterial suspension was then heated for 30 min at 65°C and centrifuged at 6,000 × *g* for 10 min. Supernatants were recovered and centrifuged at 21,000 × *g* for 30 min to remove the remaining debris. Next, ammonium sulfate (Sigma) was added to the supernatants until 60% saturation, and proteins were precipitated overnight at 4°C with stirring. Ammonium sulfate precipitates were recovered by centrifugation at 21,000 × *g* for 30 min and pellets were resuspended in 10 mL of 50 mM Tris, 10 mM NaCl. The extract was dialyzed twice in PBS (for 3 h and then overnight) at 4°C with stirring. Finally, the extract was filtered through a 0.2 μM filter (Amicon Ultra-15, Millipore), resuspended in PBS and stored at −20°C. The presence of AAF/II fimbriae was confirmed by SDS-PAGE. In order to remove contaminant endotoxins, the extract was subjected to 3 cycles of a triton X-114 treatment as previously reported (Teodorowicz et al., 2017). LPS endotoxin was detected using a Pierce *Limulus* amebocyte lysate (LAL) chromogenic endotoxin quantification kit (Thermo Scientific) according to the manufacturer's instructions. Extracted fimbrial endotoxin content was below detection level (0.125 EU/mL).

### Proteinase K Treatment

AAF/II fimbrial extract was incubated at 70°C for 10 min with 20 μg/mL Proteinase K (Qiagen) unless otherwise specified. In order to inactivate the enzyme, an EDTA-free protease inhibitor cocktail 100X (Thermo Scientific) was added to the extract and incubated for 15 min at 95°C. Proteinase K inactivation was corroborated by incubation of active and inactivated Proteinase K with BSA, followed by SDS-PAGE to evaluate protein integrity.

### Cell Assays

HT-29 and T84 cells were grown in 48-well plates (Corning). For agonist experiments, epithelial cells were incubated for 3 h with AAF/II fimbrial extract or 100 ng/mL TLR2 and TLR4 agonists BLP (InvivoGen) and LPS (*E. coli* O55:B5, Sigma), respectively. Then, the cells were washed and incubated for 3 h at 37°C under 5% CO_2_. Supernatants were collected for measuring Interleukin-8 (IL-8) levels. For EAEC infections, cells were incubated for 30 min, respectively with McCoy's or DMEM- F12 medium in the presence or absence of TLR2 and TLR4 antagonist OxPAPC (InvivoGen) or TLR4 antagonist TAK-242 (Merck), as instructed by the manufacturer. The cells were washed and incubated in medium supplemented with gentamicin (50 μg/mL) at 37°C under 5% CO_2_ for 3 h. Supernatants were then collected to measure inflammatory biomarkers.

### Inflammatory Markers Quantification

IL-8 levels were measured by ELISA as previously reported (Harrington et al., 2005). To quantify 38 cytokines, chemokines, and growth factors we used a HCYTOMAG-60K (Merck) multiplex assay kit. Supernatants from the cell were processed in a Luminex 200 instrument according to the manufacturer's instructions.

### Statistical Analysis

Experiments were performed in triplicate, and the results are expressed as the mean ± standard deviation. Statistical comparisons between groups were performed using an ANOVA, followed by Tukey's test; a *P-*value of < 0.05 was considered statistically significant. For the Luminex assay, analytes were selected as detectable using Student's *t*-test with a *P-*value < 0.05 and a 2-fold change compared to uninfected cells. The analysis was conducted using the GraphPad Prism 6 software.

## Results

### Blocking of TLR4 Reduces Induction of IL-8 Secretion in HT-29 Cells Infected With EAEC

**Figure 1:**
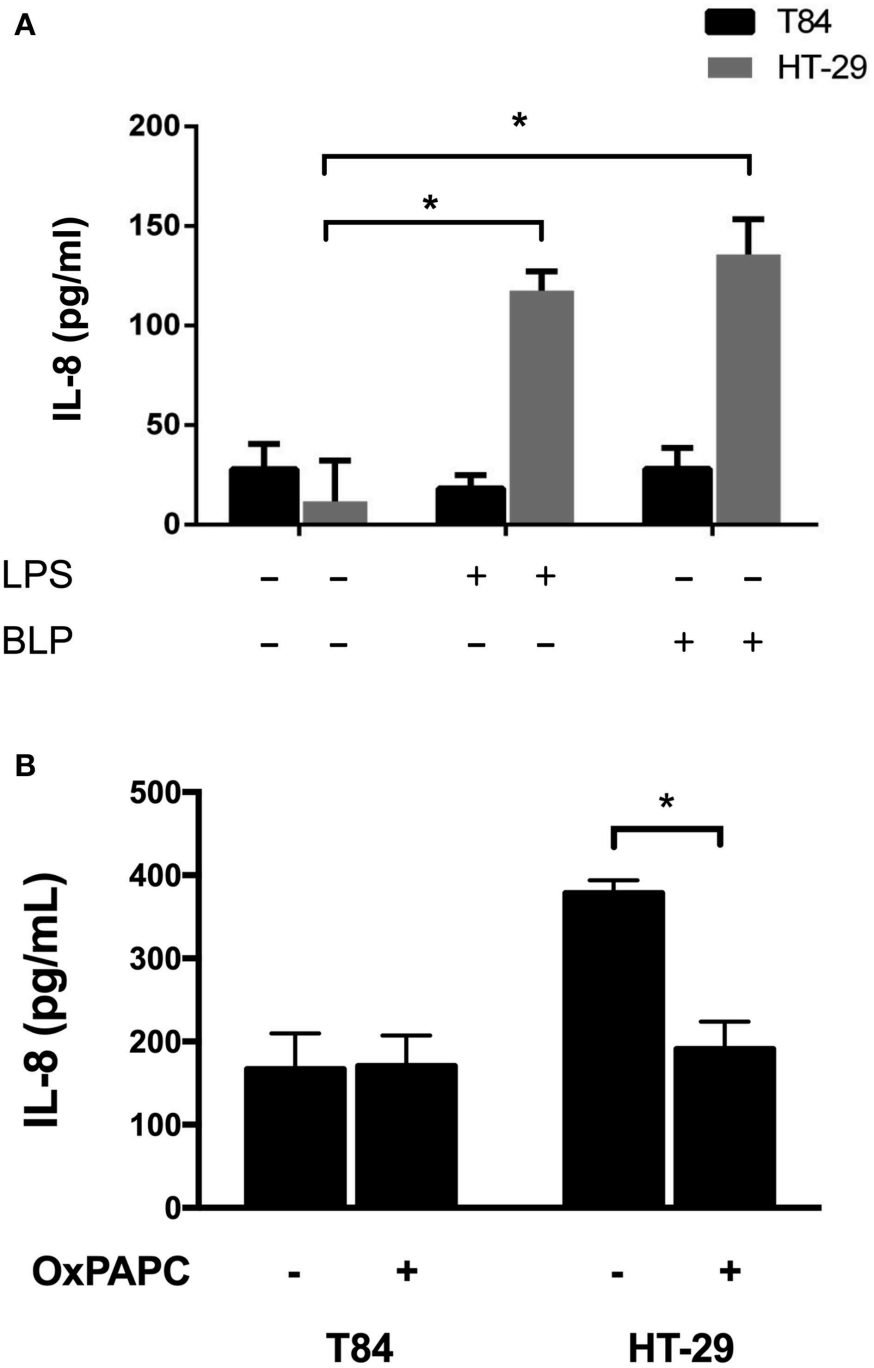
Effect of TLR2 and TLR4 agonists and antagonists on IL-8 secretion in intestinal epithelial cell lines. T84 and HT-29 cells were **(A)** incubated for 3 h with TLR2 and TLR4 agonists LBP and LPS, respectively, or **(B)** infected with EAEC strain 042 in the presence or absence of the TLR2 and TLR4 antagonist OxPAPC. IL-8 secretion was measured by ELISA. (–), vehicle control. Experiments were performed in triplicate. The bars represent the mean of three experiments + S.D. ^*^Significantly different (*p* < 0.05).

**Figure 2:**
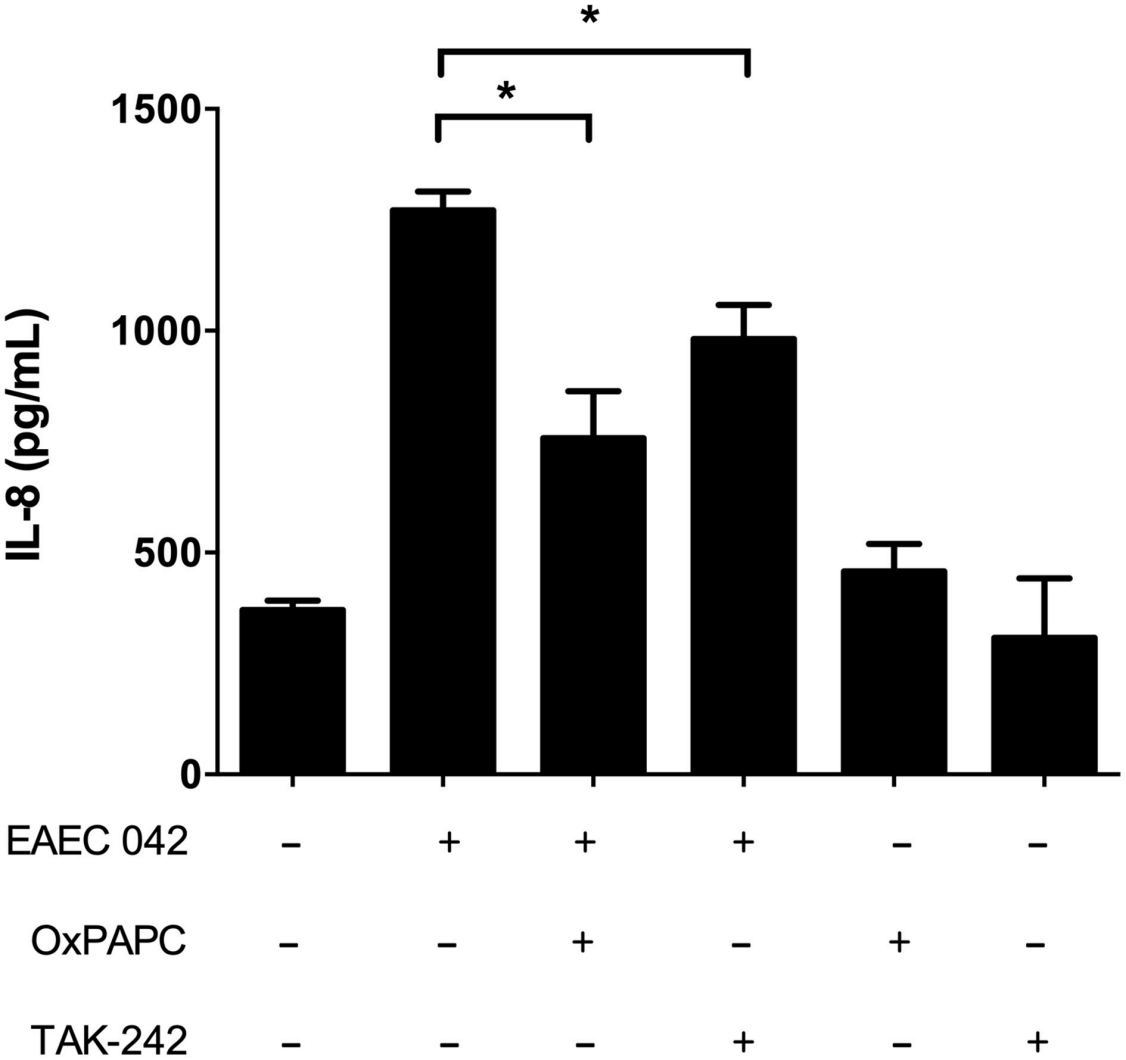
Blocking TLR4 reduces IL-8 secretion in HT-29 cells infected with EAEC strain 042. HT-29 cells were infected with EAEC strain 042 in the presence or absence of the TLR2 and TLR4 antagonist OxPAPC (30 μg/mL) or the TLR4 antagonist TAK-242 (50 ng/mL). IL-8 secretion was measured by ELISA. (–), vehicle control. Experiments were performed in triplicate. The bars represent the mean of three experiments + S.D. ^*^Significantly different (*p* < 0.05).

In order to study the participation of TLR4 in IL-8 secretion as a response to EAEC, we determined an adequate intestinal epithelial cell line as our study model. T84 and HT-29 cells were incubated with TLR2 (LBP) and TLR4 (LPS) agonists, and we found that both agonists induce IL-8 secretion in HT-29 cells, but not in T84 cells (Figure 1A). A mutual antagonist of TLR2 and TLR4 (OxPAPC) consistently reduced IL-8 levels in response to EAEC strain 042 in HT-29 cells, but had no effect on T84 cells (Figure 1B). Given these results, we used HT-29 cells and a TLR4 specific antagonist (TAK-242) to evaluate the role of TLR4 in EAEC infection. In HT-29 cells treated with TAK-242 and infected with EAEC strain 042, we found that IL-8 levels were similar to those observed when the cells were infected in the presence of the antagonist OxPAPC (Figure 2).

### AAF/II Fimbriae Induce IL-8 Secretion in HT-29 Cells Via TLR4

**Figure 3:**
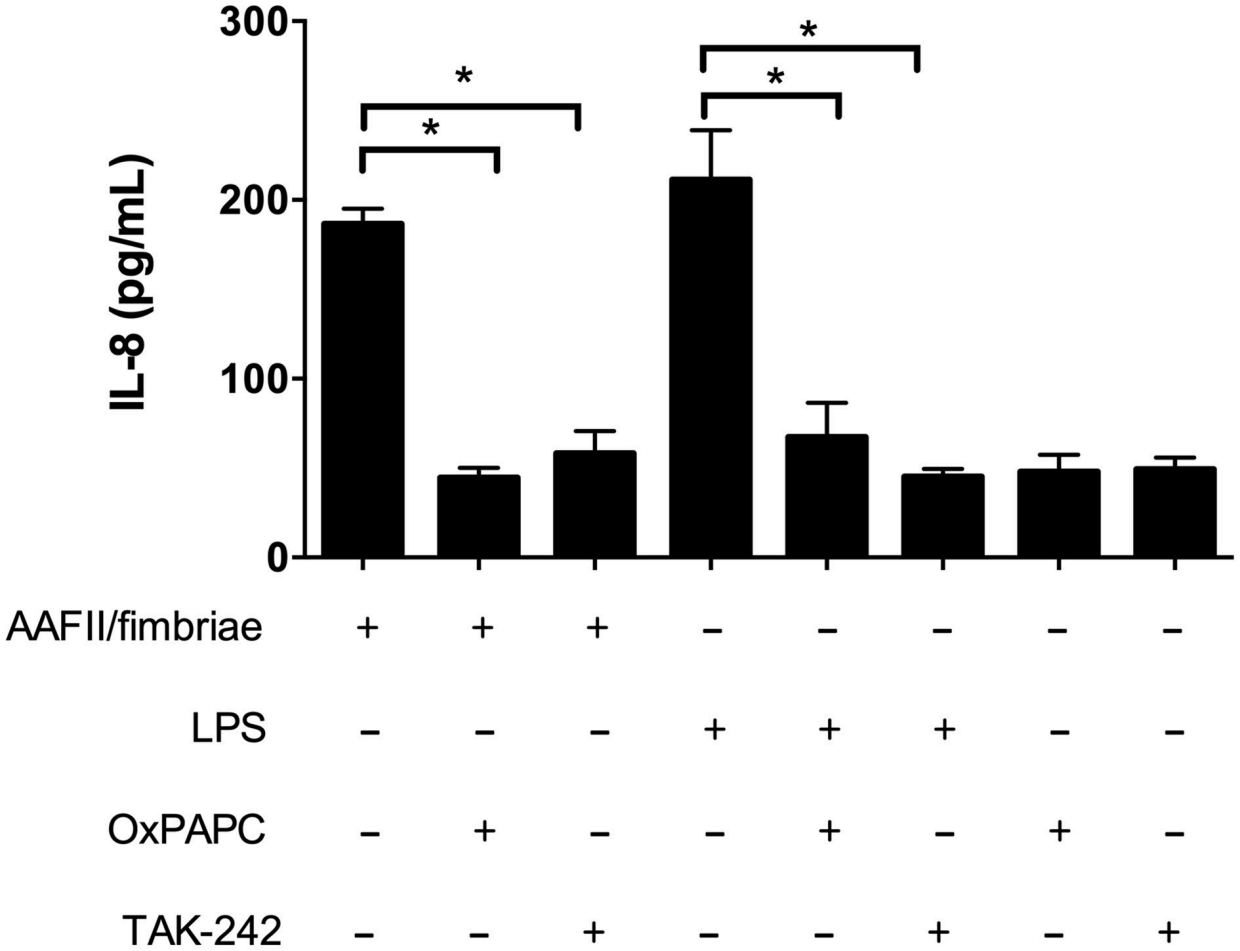
AAF/II extract induces IL-8 secretion via TLR4. HT-29 cells were incubated with an AAF/II extract in the presence or absence of the TLR2 and TLR4 antagonist OxPAPC (30 μg/mL) or the TLR4 antagonist TAK-242 (50 ng/mL). IL-8 secretion was measured by ELISA. (–), vehicle control. Experiments were performed in triplicate. The bars represent the mean of three experiments + S.D. ^*^Significantly different (*p* < 0.05).

We obtained an extract enriched in AAF/II fimbriae in order to evaluate the role of this colonization factor in the induction of IL-8 secretion in HT-29 cells. We found that incubation with the fimbrial extract induces IL-8 secretion in a dose-dependent manner, reaching a maximum of IL-8 secretion at 5 μg/ml (Supplementary Figure 1). Induction of IL-8 secretion by AAF/II fimbriae was reduced in the presence of the antagonist OxPAPC and TAK-242, suggesting that TLR4 participates in the response elicited by AAF/II (Figure 3). In addition, we subjected the extract to proteinase K treatment to assess the participation of proteins in the induction of IL-8 secretion. We observed that induction of IL-8 secretion was significantly reduced in the fimbrial extract treated with Proteinase K in a dose-dependent manner (Supplementary Figure 2).

### TLR4 Participates in the Global Inflammatory Response Induced by AAF/II Fimbriae on Intestinal Epithelial Cells

**Figure 4:**
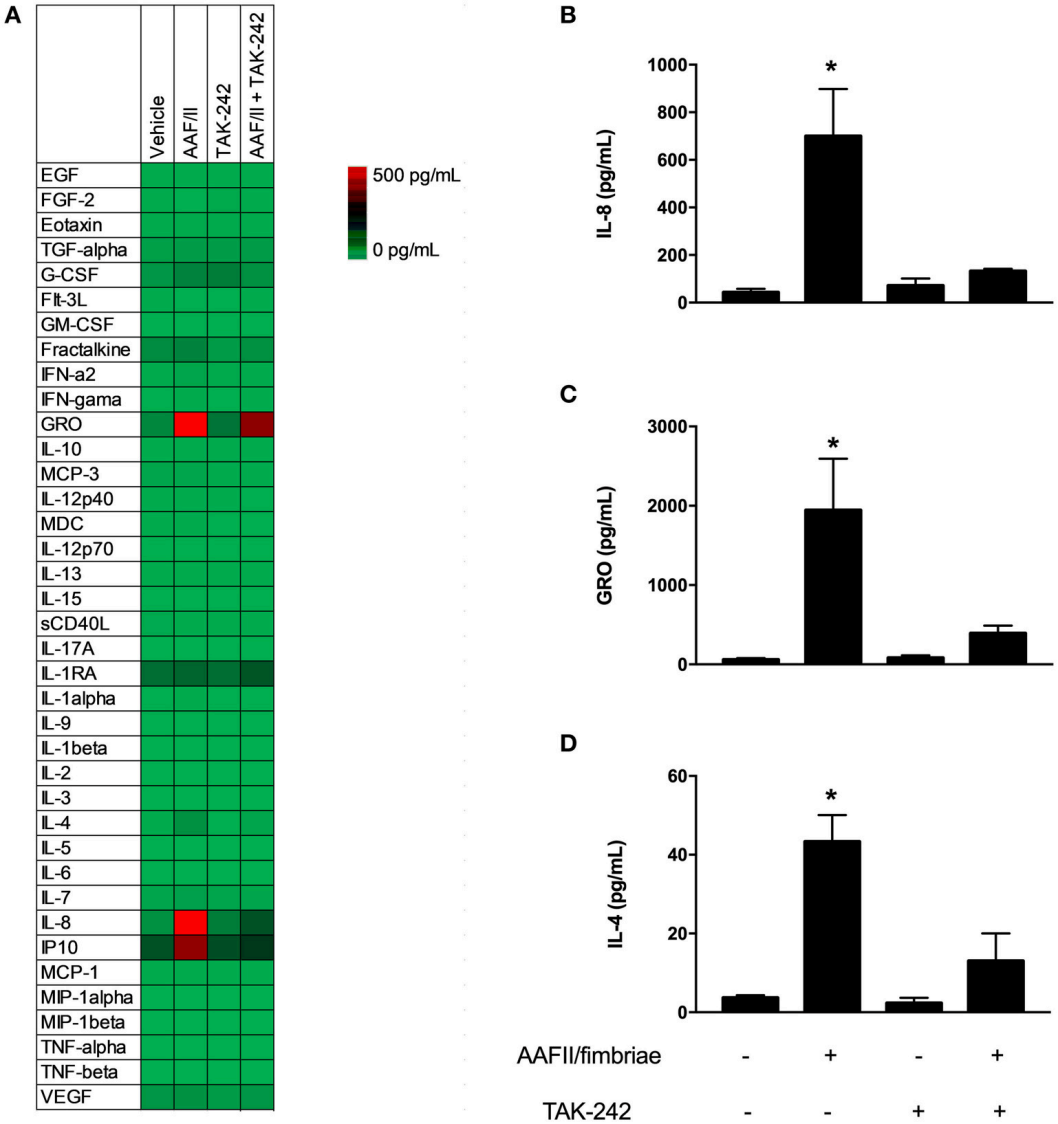
AAF/II fimbriae induce secretion of cytokines and chemokines. HT-29 cells were stimulated with AAF/II fimbriae extract 5 μg/mL for 3 h in the presence or absence of TLR4 antagonist TAK-242, followed by 3 h without stimuli. Media were collected and cytokines, chemokines and growth factors were measured through a multiplex Luminex™ assay. **(A)** Heat map of 38 analytes measured. **(B–D)** Three analytes with higher induction by AAF/II. (–), vehicle control. Experiments were performed in triplicate. The bars represent the mean of three experiments + S.D. ^*^Significantly different (*p* < 0.05).

First, we quantified 38 cytokines, chemokines and growth factors related to inflammation in cells infected with EAEC 042 and cells incubated with AAF/II fimbriae (Table 1). EAEC strain 042 induce the secretion of granulocyte colony-stimulating factor (G-CSF), granulocyte-monocyte colony stimulating factor (GM-CSF), growth-regulated oncogene (GRO), monocyte chemotactic protein-3 (MCP3), macrophage-derived chemokine (MDC), IL-4, IL-8, interferon gamma-induced protein 10 (IP-10) and tumor necrosis factor (TNF)-α. Of these detectable markers only GRO, IL-4 and IL-8 were induced by the AAF/II fimbriae. All these markers were significantly reduced when cells were treated with the AAF/II fimbriae and TAK-242 compared to cells treated with the AAF/II fimbriae alone (Figure 4).

**Table 1:** Detectable and non-detectable cytokines, chemokines, and growth factors determined using a 38-multiplex assay in HT-29 cells infected with EAEC 042 or incubated with AAF/II extract.

	**EAEC 042**	**AAF/II fimbriae**
Detectable cytokines, chemokines and growth factors	G-CSF, GM-CSF, GRO, MCP-3, MDC, IL-4, IL-8, IP-10, TNF-α	GRO, IL-4, IL-8
Non-detectable cytokines, chemokines and growth factors	EGF, FGF-2, Eotaxin, TGF-α, Flt-3L, Fractalkine, IFN-α2, IFN-γ, IL-10, IL-12p40, IL-12p70, IL-13, IL-15, sCD40L, IL-17A, IL-1RA, IL-1α, IL-9, IL-1β, IL-2, IL-3, IL-5, IL-6, MCP-1, MIP-1α, IL-7, MIP-1β, TNF-β, VEGF	EGF, FGF-2, Eotaxin, TGF-α, G-CSF, Flt-3L, GM-CSF, Fractalkine, IFN-α2, IFN-γ, IL-10, MCP-3, IL-12p40, MDC, IL-12p70, IL-13, IL-15, sCD40L, IL-17A, IL-1RA, IL-1α, IL-9, IL-1β, IL-2, IL-3, IL-5, IL-6, IP-10, MCP-1, MIP-1α, IL-7, MIP-1β, TNF-α, TNF-β, VEGF

## Discussion

EAEC infections are a public health problem worldwide, since they cause persistent and acute diarrhea in both industrialized and developing countries (Huang et al., 2007; Hebbelstrup et al., 2014). Infections with this pathogen share some distinct hallmarks, such as persistent diarrhea and intestinal inflammation (Huang and Dupont, 2004; Boll et al., 2012). EAEC infection has also been associated with growth impairment in children, regardless of the presence of diarrhea (Guerrant et al., 2008). Growth impairment, along with other factors, has been linked to the inflammatory response that is triggered in response to this pathogen. This inflammatory response is characterized by increased levels of fecal lactoferrin and cytokines such as IL-1β and IL-8 (Steiner et al., 2000; Greenberg et al., 2002). Using epithelial cell models, EAEC infection induces the secretion of several inflammatory markers, with IL-8 being a key marker of the EAEC-induce inflammation in epithelial cells (Harrington et al., 2005; Cennimo et al., 2009; Sanchez-Villamil et al., 2016). However, neither the bacterial nor the intestinal factors implicated in the orchestration of the inflammatory response due to EAEC have been fully clarified.

Toll-like receptors are a family of immune activators that recognize PAMPs from different pathogens. Two members of this family, TLR2 and TLR4, have been implicated in the recognition of fimbriae from various pathogens, while TLR5 recognizes flagellin. Once activated, these receptors induce intracellular signaling cascades that culminate in NF-κB activation and the secretion of proinflammatory cytokines (Hug et al., 2018). In this work, we found evidence suggesting that TLR4 participates in the inflammatory response developed against EAEC.

Reports of TLR2 and TLR4 expression and their response to agonists in intestinal epithelial cell lines are controversial (Abreu et al., 2001; Abreu, 2003; Melmed et al., 2003; Uehara et al., 2007). We selected intestinal epithelial cell lines T84 and HT-29 to test as our study models. T84 is the model most frequently employed when studying EAEC infection, and it has been reported that HT-29 cells secrete IL-8 in the presence of TLR2 and TLR4 agonists (Uehara et al., 2007; Strauman et al., 2010). We found that TLR2 and TLR4 agonists LBP and LPS induce IL-8 secretion in HT-29 cells, but not in T84 cells (Figure 1A). These observations are in agreement with previous reports (Schuerer-Maly et al., 1994) and may be explained by the nature of the intestinal epithelial cell lines tested here, as the intestinal epithelium presents a differentiation gradient, and it has been shown that HT-29 cells are similar to the undifferentiated bottom of intestinal crypt cells (Rousset, 1986), which is where TLR2 and TLR4 are expressed. In contrast, T84 cells resemble a more luminal differentiated state, where these receptors have a lower presence (Furrie et al., 2005).

OxPAPC is a mixture of the phospholipid PAPC subjected to oxidation and it specifically inhibits TLR2 and TLR4 by competing with co-receptors required for activation, such as CD14, LBP and MD2 (Erridge et al., 2008). We found that incubation with the mutual antagonist OxPAPC decreased IL-8 levels in response to EAEC strain 042 in HT-29 cells compared to cells infected in absence of OxPAPC (Figure 1B). To elucidate the individual contribution of TLR2 and TLR4, HT-29 cells were infected with EAEC strain 042 in the presence of the TLR4 specific antagonist TAK-242. We observed that TAK-242 presence decreases IL-8 secretion, as observed when cells were infected in the presence of OxPAPC (Figure 2), implicating TLR4 in the inflammatory response against EAEC strain 042. It is important to note that IL-8 secretion was not completely eliminated in the presence of OxPAPC and TAK-242, a situation that might be explained by the action of another bacterial surface protein, such as flagellin, which has been shown to induce IL-8 secretion through TLR5 (Khan et al., 2004).

The most distinctive colonization factor of EAEC strain 042 is the AAF/II fimbria, which participates throughout the infection cycle of this bacterium. This fimbria enhances adhesion to the intestinal epithelium by interacting with multiple extracellular matrix proteins, such as laminin, collagen, cytokeratin-8 and fibronectin (Farfan et al., 2008; Izquierdo et al., 2014b). Previously, we reported that the mechanism of fibronectin-mediated EAEC strain 042 adhesion occurs by binding to integrin α5β1, thereby bridging the bacterial fimbria to a transmembrane protein (Izquierdo et al., 2014a). Likewise, AAF/II has been implicated in biofilm formation and induces IL-8 secretion and PMN infiltration in intestinal epithelial cell cultures and in human intestinal xenografts *in vivo*, a process mediated by epithelial transmembrane mucin MUC1 (Boll et al., 2017). We hypothesized that AAF/II could be a novel TLR4 agonist that induces IL-8 secretion in HT-29 cells. To test this hypothesis, we obtained an AAF/II-enriched extract from an EAEC *fliC* mutant to discard the effect of flagella and the TLR5 receptor. We determined that cells incubated with this extract have increased levels of IL-8 that can be blocked in the presence of OxPAPC and TAK-242 (Figure 3). These results position AAF/II as a TLR4 agonist. Nonetheless, the AAF/II-enriched extracts may contain traces of endotoxin, which could account for the induction of IL-8 secretion as described in *Porphyromonas gingivalis* fimbriae (Nozoe et al., 2017). To avoid this, we subjected the fimbrial extract to two different treatments to remove traces of LPS below the detection limit. On the other hand, we determined that cells incubated with the fimbrial extract treated with Proteinase K had lower IL-8 levels than cells incubated with the AAF/II extract and that IL-8 levels decreased in a Proteinase K dose-dependent manner (Supplementary Figure 2). Together, our results implicate both AAF/II and TLR4 as contributors in IL-8 secretion in response to EAEC strain 042.

Intestinal inflammation is a major hallmark of EAEC infection, characterized by high levels of secreted IL-8. IL-8 plays a pivotal role in the intestinal inflammation and recruitment of neutrophils during enteric infection by several enteropathogens, including EAEC. In addition to IL-8, several proinflammatory markers have been associated with EAEC infection (Harrington et al., 2005). In this work, we quantified 38 cytokines, chemokines, and growth factors related to inflammation. Several markers were found significantly present in cells infected with EAEC strain 042 compared to uninfected cells, some of them in agreement with previous reports (Cennimo et al., 2009). In addition to IL-8, we found that GRO and IL-4 were also present in cells incubated with the AAF/II fimbriae. All these markers decreased significantly when cells were treated with AAF/II in the presence of TLR4 antagonist (Figure 4). GRO is a chemokine involved in the recruitment of neutrophils in several bacterial and non-bacterial infections including *H. pylori* (Tran et al., 2017), *E. coli* (Lee et al., 2018), *B. cereus* (Coburn et al., 2018), rhinovirus (Jamieson et al., 2019) and *Leishmania* (Ronet et al., 2018). Moreover, it has been shown to be associated with neutrophil recruitment mediated by the Th17 response (Fujie et al., 2012). In TLR4^−/−^ knockout mice, gene expression of the GRO encoding gene was significantly reduced after infection with *B. cereus* (Coburn et al., 2018), supporting the role of TLR4 receptor in the expression of GRO in response to bacterial infections. For EAEC, GRO gene expression was induced on intestinal epithelial cells as part of the inflammatory response against this pathogen (Harrington et al., 2005; Prata et al., 2018). Overall, our data support both the involvement of GRO in the immune response to EAEC by epithelial cells and the participation of TLR4 receptor in this response.

IL-4 is one of the main cytokines released during Th2 cell responses, and it is known to stimulate B-cell proliferation, IgG and IgE production, mast cell proliferation (Lundgren et al., 1989; Turner et al., 2014; McLeod et al., 2015) and mucin production from epithelial cells (Iwashita et al., 2003), among other effects required for a fine immune response against pathogens. Although IL-4 has been shown to reduce bacterial clearance in acute infection (Song et al., 2015); it is necessary for correct tissue repair (Salmon-Ehr et al., 2000), making it an important cytokine after bacterial clearance. Given our results, EAEC infection (and AAF/II fimbriae stimuli) on epithelial cells might promote the immune response to shift toward a Th1 response, although Th2 and anti-inflammatory response might also be present by IL-4 induction, as has been shown to occur in other immune cell models where pro- and anti-inflammatory cytokines are induced by the same pathogen (Kamada et al., 2008; Kitani et al., 2014). It is important to note that the presence of this cytokine in response to EAEC infection was evaluated previously, but it was not detected in the supernatant of infected cells (Cennimo et al., 2009). A possible explanation for these results might be associated with the cell lines tested (HCT-8). Considering the importance of IL-4 in the inflammatory process by pathogens, the role of these cytokines in the context of the EAEC pathogenesis requires further investigation.

Our study has limitations. We tested two intestinal epithelial cell lines, T84 and HT-29, and only HT-29 cells respond to TLR4 agonist. Although HT-29 cells have been extensively used to study EAEC pathogenesis as well as other enteropathogens, the observations described here require further validation through other *in vitro* models of infection. Unfortunately, there is no a reproducible and reliable animal model to fully characterize the immunological response to EAEC in the intestinal mucosa (Philipson et al., 2013), but the development of an organotypic culture system might help to corroborate our findings.

Our work provides new insights into the inflammatory response elicited by EAEC strain 042. Our results implicate TLR4 in the induction of IL-8 secretion in response to EAEC strain 042 in intestinal epithelial cells and suggest that AAF/II is one of the factors mediating this response.

## Author Contributions

AA participated in the design of the study, fimbrial extraction, infection assay, IL-8 measurement, samples processing, data analysis and writing of the manuscript. MO-M carried out the infection assay, Luminex measurement, IL-8 measurement, sample processing, data analysis and writing of the manuscript. EM carried out the fimbrial extraction and infection assay. RS carried out the fimbrial extraction. JN participated in the manuscript writing and final approval of the manuscript. FR-P participated in the manuscript writing and final approval of the manuscript. MF participated in the design of the study, data analysis, manuscript writing and final approval of the manuscript. All authors read and approved the final manuscript.

### Conflict of Interest Statement

The authors declare that the research was conducted in the absence of any commercial or financial relationships that could be construed as a potential conflict of interest.

## Supplementary Material

Supplementary Figure 1 HT-29 cells were incubated with increasing concentrations of AAF/II fimbriae extract. IL-8 secretion was measured by ELISA. (–), vehicle control. Experiments were performed in triplicate. The bars represent the mean of three experiments + S.D. ^*^Significantly different (*p* < 0.05).

Supplementary Figure 2 HT-29 cells were incubated with an AAF/II extract treated with increasing concentrations of Proteinase K. IL-8 secretion was measured by ELISA. (–), vehicle control. Experiments were performed in triplicate. The bars represent the mean of three experiments + S.D. ^*^Significantly different (*p* < 0.05).
