# Supplementary figures and images for: TLR4 Participates in the Inflammatory Response Induced by the AAF/II Fimbriae From Enteroaggregative Escherichia coli on Intestinal Epithelial Cells
Source: Front Cell Infect Microbiol. 2019 May 3;9:143. doi: 10.3389/fcimb.2019.00143 (PMC6509964; doi:10.3389/fcimb.2019.00143)

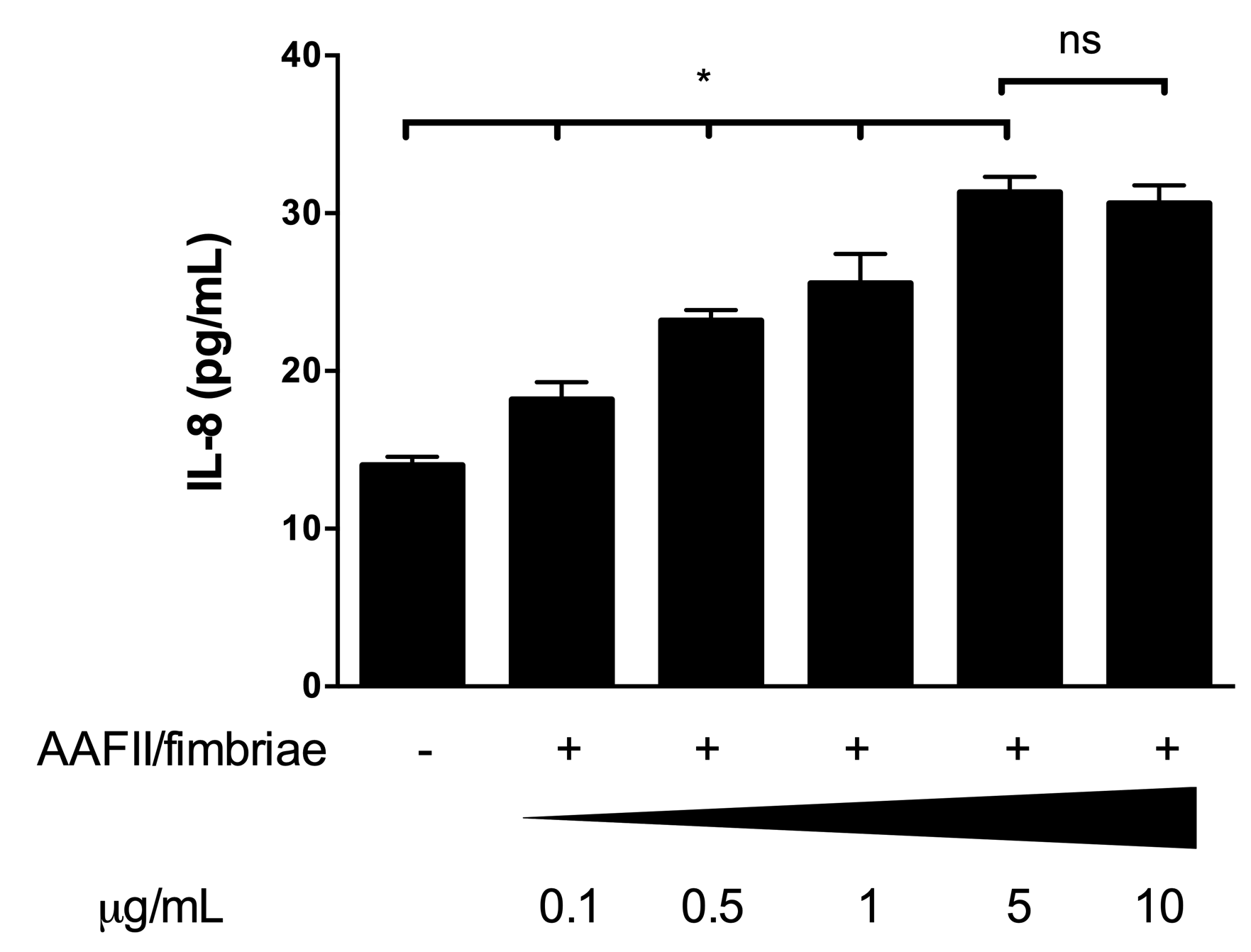

Supplement: Supplementary Figure 1 — HT-29 cells were incubated with increasing concentrations of AAF/II fimbriae extract. IL-8 secretion was measured by ELISA. (–), vehicle control. Experiments were performed in triplicate. The bars represent the mean of three experiments + S.D. *Significantly different (p < 0.05). [file Image_1.TIFF]

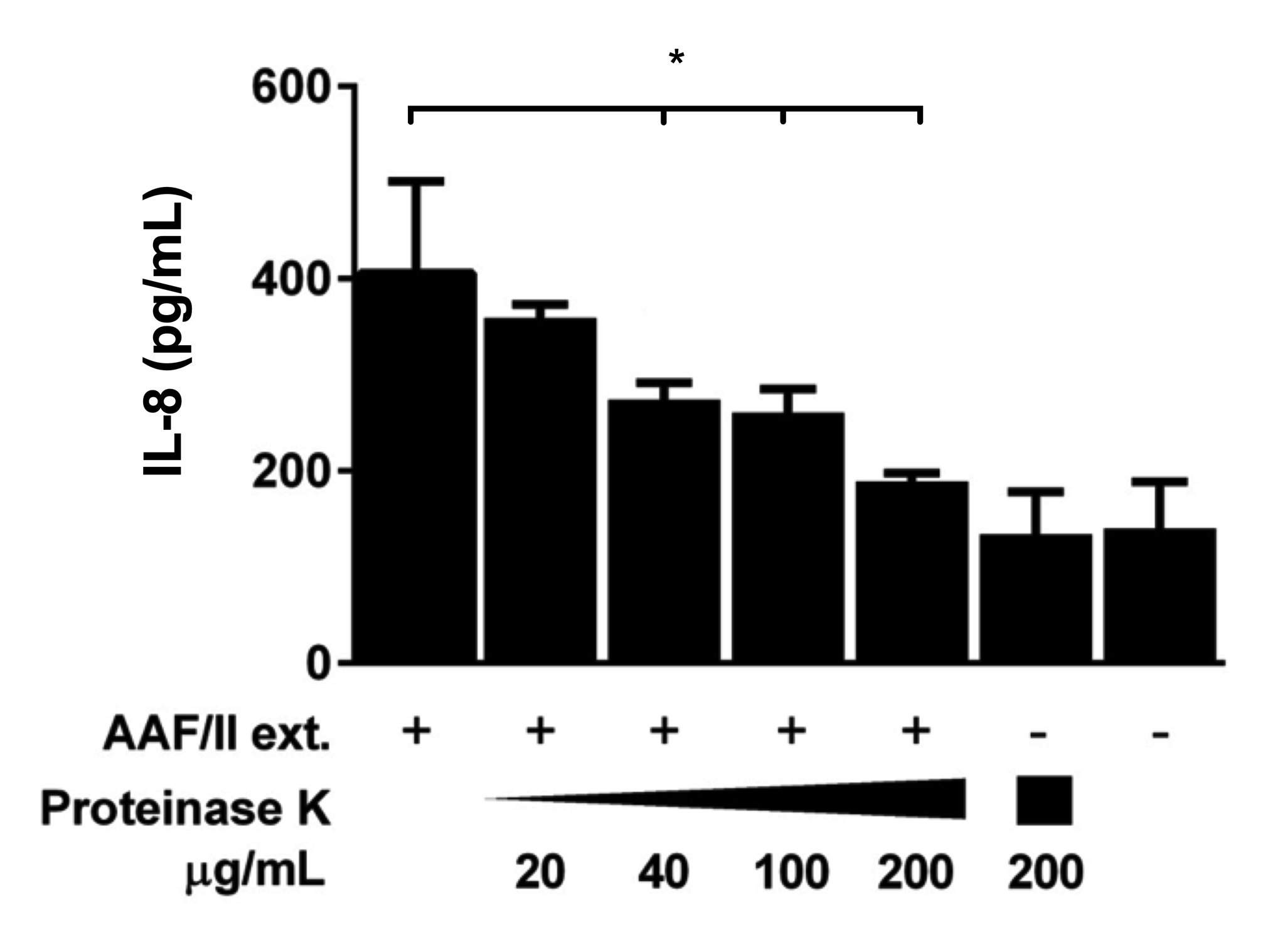

Supplement: Supplementary Figure 2 — HT-29 cells were incubated with an AAF/II extract treated with increasing concentrations of Proteinase K. IL-8 secretion was measured by ELISA. (–), vehicle control. Experiments were performed in triplicate. The bars represent the mean of three experiments + S.D. *Significantly different (p < 0.05). [file Image_2.TIFF]
